# 6-OHDA-Induced Changes in Parkinson`s Disease-Related Gene Expression are not Affected by the Overexpression of PGAM5 in In Vitro Differentiated Embryonic Mesencephalic Cells

**DOI:** 10.1007/s10571-015-0207-5

**Published:** 2015-05-19

**Authors:** Tomasz Maciej Stępkowski, Iwona Wasyk, Agnieszka Grzelak, Marcin Kruszewski

**Affiliations:** Centre for Radiobiology and Biological Dosimetry, Institute of Nuclear Chemistry and Technology, Dorodna 16, 03-195 Warsaw, Poland; Department of Molecular Biophysics, University of Łódź, Banacha 12/16, 90-237 Łódź, Poland; Department of Molecular Biology and Translational Research, Institute of Rural Health, Jaczewskiego 2, 20-090 Lublin, Poland; Department of Medical Biology and Translational Research, Faculty of Medicine, University of Information Technology and Management, Sucharskiego 2, 35-225 Rzeszów, Poland

**Keywords:** Parkinson’s disease, Gene expression, 6-hydroxydopamine, LUHMES cells, PGAM5

## Abstract

LUHMES cells, a recently established line of immortalized embryonic mesencephalic cells, are the novel in vitro model for studying Parkinson’s disease (PD) and dopaminergic neuron biology. Phosphoglyceromutase 5 (PGAM5) is a mitochondrial protein involved in mitophagy, mitochondria dynamics, and other processes important for PD pathogenesis. We tested the impact of lentiviral overexpression of PGAM5 protein in LUHMES cells on their differentiation and expression of 84 PD-related genes. LUHMES cells were transduced with PGAM5 or mock and treated with 100 μM 6-hydroxydopamine (6-OHDA), a model PD neurotoxin. Real-Time PCR analysis revealed that the treatment with 6-OHDA-induced changes in expression of 44 PD-related genes. PGAM5 transduction alone did not cause alternations in PD-related genes expression, nor it affected changes in gene expression mediated by 6-OHDA. The 6-OHDA-induced PD-related gene expression profile of LUHMES cells is presented for the first time and widely discussed.

**Electronic supplementary material:**

The online version of this article (doi:10.1007/s10571-015-0207-5) contains supplementary material, which is available to authorized users.

## Introduction

Parkinson`s disease (PD) is the second most common neurodegenerative disorder affecting mainly elderly people. Till know, the detailed mechanism responsible for its development remains unknown. The presently used therapies are mainly symptomatic and alleviate only a part of the symptoms that dramatically deteriorate the quality of life of the patients. Taking to account the aging of the population of many developed countries and enormous socio-economical burden caused by PD, research regarding the disease are considered as one of the main healthcare priorities for the society (Dexter and Jenner 2013).

This study was aimed at evaluating the effects of phosphoglyceromutase 5 (PGAM5) overexpression in the cellular model of Parkinson`s disease, 6-hydroxydopamine (6-OHDA)-treated differentiated human embryonic mesencephalic cells of the Lund Human Mesencephalic (LUHMES) cell line. PGAM5 is a recently discovered mitochondrial protein that may be strongly implicated in the molecular processes responsible for the development of PD. It was discovered as a regulator of the NRF2/KEAP1 pathway which is the main pathway responsible for triggering transcription of a battery of antioxidant and pro-survival genes in response to oxidative and electrophile stressors (Lo and Hannink 2008; Venugopal and Jaiswal 1996). The NRF2/KEAP1 pathway is responsible for the alleviation of the effects of PD model toxins, both in vitro and in vivo (Chen et al. 2009; Cuadrado et al. 2009; Lee et al. 2003; Shin 2014). Moreover, the neuroprotective effects of the so-called “chemopreventive natural dietary compounds”, such as curcumin, resveratrol, or sulforaphan, are also mediated by the NRF2/KEAP1 signaling pathway. PGAM5 was found to bind the antiapoptic protein Bcl-xL and regulate its KEAP1-dependent ubiquitination and subsequent degradation (Lo and Hannink 2006). On the other hand, PGAM5 itself is a KEAP1 target and tethers the whole NRF2-KEAP1 complex to the mitochondrion. Silencing the PGAM5 increased the NRF2-dependent transcription in HeLa cells (Lo and Hannink 2008). It is plausible to assume that PGAM5 might be responsible for the fine tuning of the NRF2/KEAP1-dependent apoptic or survival response during oxidative stress (Stepkowski and Kruszewski 2011). It was recently shown that PGAM5 indirectly mediated Bcl-xL degradation in irradiated prostate cancer cells and this process was responsible for their radiosensitization by parthenolide. Interestingly, in normal cells the same compound protected against cell death by activating NRF2-dependent transcription (Xu et al. 2013).

PGAM5 is also responsible for dephosphorylating and activating ASK1, a higher order MAP kinase responsible for induction of neuronal apoptosis via p38- JNK axis in 6-OHDA and 1-methyl-4-phenyl-1,2,3,6-tetrahydropyridine (MPTP) models of PD (Ouyang and Shen 2006; Takeda et al. 2009).

Finally, the importance of PGAM5 in mechanisms involved in the development of PD was directly shown by Lu et al. (2014), who found out that knockout of PGAM5 in mouse cause a Parkinson`s like movement disorder. This pathogenic state was caused by the impairment of PINK1-mediated mitophagy. The importance of PGAM5 in PINK1/PARKIN-mediated mitophagy is also well supported by the previous in vitro studies (Sekine et al. 2012).

In this work, we used one of the most advanced in vitro models of PD, namely differentiated LUHMES cells treated with 6-OHDA (Lotharius et al. 2005; Schildknecht et al. 2009). Unlike commonly used neuroblastoma cell lines: SH-SY5Y or BE2-M17, LUHMES cells differentiation is imprinted intrinsically rather than induced by external factors, e.g., retinoic acid. The LUHMES cells are kept in undifferentiated, proliferating stage by the expression of v-myc oncogene under control of Tet element promoter that is silenced in the presence of tetracyclin. Addition of the antibiotic and ablation of the fibroblast growth factor from the medium lead to differentiation of the cells into post-mitotic neurons phenotypically similar to mature human dopaminergic neurons. 6-OHDA is a pro-oxidative neurotoxin commonly used to model PD in rodents. The intrastriatal administration of 6-OHDA in rodents creates pathological events similar to human PD: formation of aggregated alpha synuclein bodies, proteasome inhibition, increased ubiquitination, nitration and oxidation of proteins, gluthathione depletion, and caspase 3 cleavage (Blesa et al. 2012). 6-OHDA-mediated effects are not dependent on a direct inhibition of mitochondrial electron transport chain, but rather by the early oxidative stress events that can be alleviated by the action of NRF2/KEAP1 pathway, and therefore possibly modified by alternating levels of PGAM5 (Cao et al. 2005). To find out whether increased cellular abundance of PGAM5 protein change the effects of 6-OHDA exposure, we analyzed the expression of 86 PD-related genes in lentiviral transduced LUHMES cells.

## Materials and Methods

### Cell Culture

LUHMES cells were grown in monolyer on NunclonΔ™ cell culture flasks coated with poly-l-ornithine (50 μg/mL) and human plasma fibronectin (1 μg/mL) (Sigma-Aldrich). Filter sterilized poly-l-ornithine was mixed with fibronectin, poured directly to the culture flasks (1 mL per 10 cm^2^) and left overnight at 37 °C. The coating mixture was then aspirated and the flasks were dried in the laminar hood. Freshly coated flasks were used in each experiment.

Undifferentiated, proliferating cells were grown in Advanced DMEM/F-12 medium (Life Technologies) supplemented with N-1 supplement (Sigma-Aldrich, N6530), basic fibroblast growth factor (bFGF) (Life Technologies) and 2 mM l-glutamine. Differentiation was conducted according to the previously established two-step protocol (Scholz et al. 2011). Briefly, when cells reached 70 % confluency medium was changed to a differentiation medium containing 1 μg/mL tetracycline instead of bFGF. On the next day (attributed as Day 2 of differentiation) cells were trypsinized and seeded to the new flasks to create a monolayer of separated differentiated cells without visible clumps of unequally differentiated cells. Cells were differentiated for 7 days before performing the final experiment.

### Lentiviral Transduction

Lentiviral plasmids were designed in the Centre for Radiobiology and Biological Dosimetry to allow simultaneous expression of mitochondrially targeted DsRed2 and long (289 amino acids) isoform of PGAM5 (CCDS53845.1) (further referred as PGAM5_L). Two lentiviral constructs built on the pLV.Des2d.P_mito-DsRed2 backbone were used in this study: PGAM5_L cDNA plasmid and control mock plasmid containing MCS and 3* FLAG sequence instead of PGAM5_L sequence. Both plasmids contained mtDsRed2 under the control of P1 promoter. PGAM5_L transcription was driven by the EF1 alpha promoter. The detailed maps of the viral vectors used in this study are provided as supplementary material (Supplementary Fig. 1). Lentiviruses packaging was outsourced (CYAGEN biosciences). On the first day of LUHMES cells differentiation lentiviral particles were added to the differentiation medium supplemented with 4 μg/mL Polybrene (10^6^ viral particles per 1 mL). After overnight transduction medium was removed and the cells were subcultured into 21 cm^2^ flasks for further differentiation for 6 days.

**Fig. 1:**
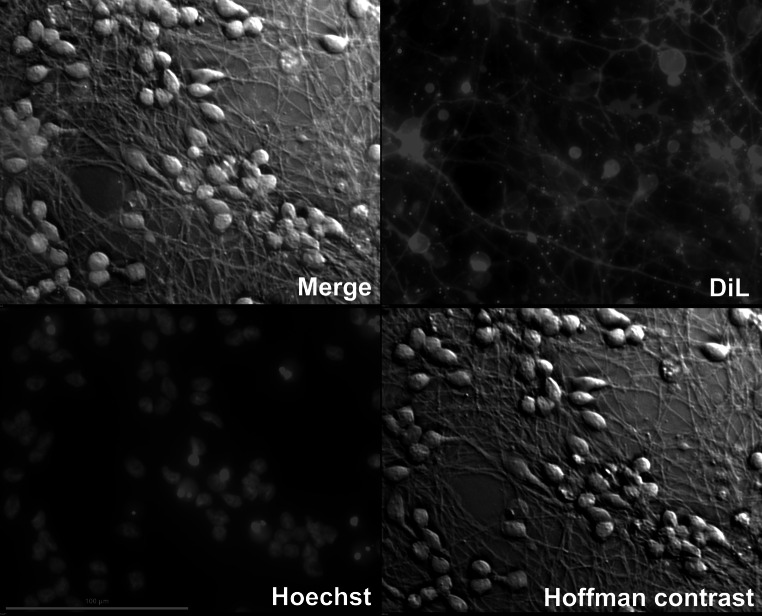
The morphology of differentiated LUHMES cells. Lipofilic dye DiL was used to visualize the neurites. Nuclei were stained with Hoechst dye and cell bodies were observed in bright field with Hoffman modulation contrast. All pictures were taken at ×40 magnitude

### 6-Hydroxydopamine Treatment

6-Hydroxydopamine hydrochloride (Sigma-Aldrich H4381) was dissolved in 1 % ascorbic acid before every experiment and protected from light. Fresh 6-OHDA solution was added directly to culture medium for 6 h, to achieve the final concentration 100 μM. Control cells were treated with the appropriate vehicle buffer.

### Microscopy Imaging

All pictures were taken with NIKON A1 fluorescence microscope equipped with NIS ELEMENTS software. NIKON 20× and 40× plan fluor objectives were used. Live cell imaging was done with the use of Hoffman modulation contrast, which is compatible with plastic cell culture dishes. Nuclei were stained with Hoechst 33342 in concentration of 2 μg/mL. To visualize neurite net, we used a lipofilic dye, Dil Stain (1,1′-dioctadecyl-3,3,3′,3′-tetramethylindocarbocyanine perchlorate, Molecular Probes).

### RNA Isolation, Reverse Transcription, and Real-Time PCR

Total RNA was extracted from the cell pellets using ReliaPrep™ mini prep system (Promega), according to the manufacturer’s protocol. RNA concentration and purity were assessed photometrically using A260/A280 ratio. RNA integrity was tested by agarose gel electrophoresis. 700 ng of the total RNA were converted to cDNA using RT^2^ First Strand Kit (SABiosciences). The cDNA was used for the expression profiling by the Human Parkinson`s RT^2^ Profiler PCR Array (SABiosciences, cat. no. PAHS-124 Z), according to the manufacturer’s instructions. This array allows for measurement of the expression of 84-genes, directly or potentially related to the PD (involved in Parkin pathway, cell adhesion, ubiquitin pathway, apoptosis, and inflammation, mitochondria metabolism, neuronal signaling, cytoskeletal organization, and genes related to the ion transport). For detailed list of genes see the manufacturer webpage, http://www.sabiosciences.com. cDNA amount corresponding to 6.35 ng of the total RNA was used for a single well reaction, to achieve an average C_t_ of 23. Relative gene expression was calculated using the ΔΔC_t_ method with HPRT1, GAPDH, and B2M genes, as the reference controls. Calculations were done using RT^2^ Profiler PCR Array Data Analysis Template provided by SABiosciences. Statistical differences were examined by Student’s t test with *p* < *0.02* considered to be statistically significant. Overexpression of *PGAM5_L* mRNA was additionally tested by Real-Time PCR using a Taqman^®^ probe specific for all PGAM5 isoforms according to a manufacturer protocol (Life Technologies).

### Western Blot Analysis

The protein concentration was determined using the Bradford method. 30 μg of cell lysates (1 % Nonidet buffer) were boiled for 10 min at 60 °C in Laemmli sample buffer. After that samples were loaded on 12 % SDS-PAGE polyacrylamide gel and separated. After protein transfer to PVDF membranes (BioRad), membranes were blocked for 1 h with 5 % milk at 4 °C and incubated with primary antibodies diluted in blocking solution overnight (4 °C). Primary antibodies (Abcam 126534) were then detected with HRP-conjugated secondary antibodies and chemiluminescence was triggered by Western Bright ECL reagent (Advansta) as substrate, and recorded on CL-XPosure films (Pierce). Blots and films were scanned. Molecular weight of protein bands was estimated in comparison to BenchMark Prestained protein ladder (Invitrogen).

## Results

### Differentiation and Morphology of Mature, Post-mitotic Neurons is not Affected by PGAM5_L Overexpression

**Fig. 2:**
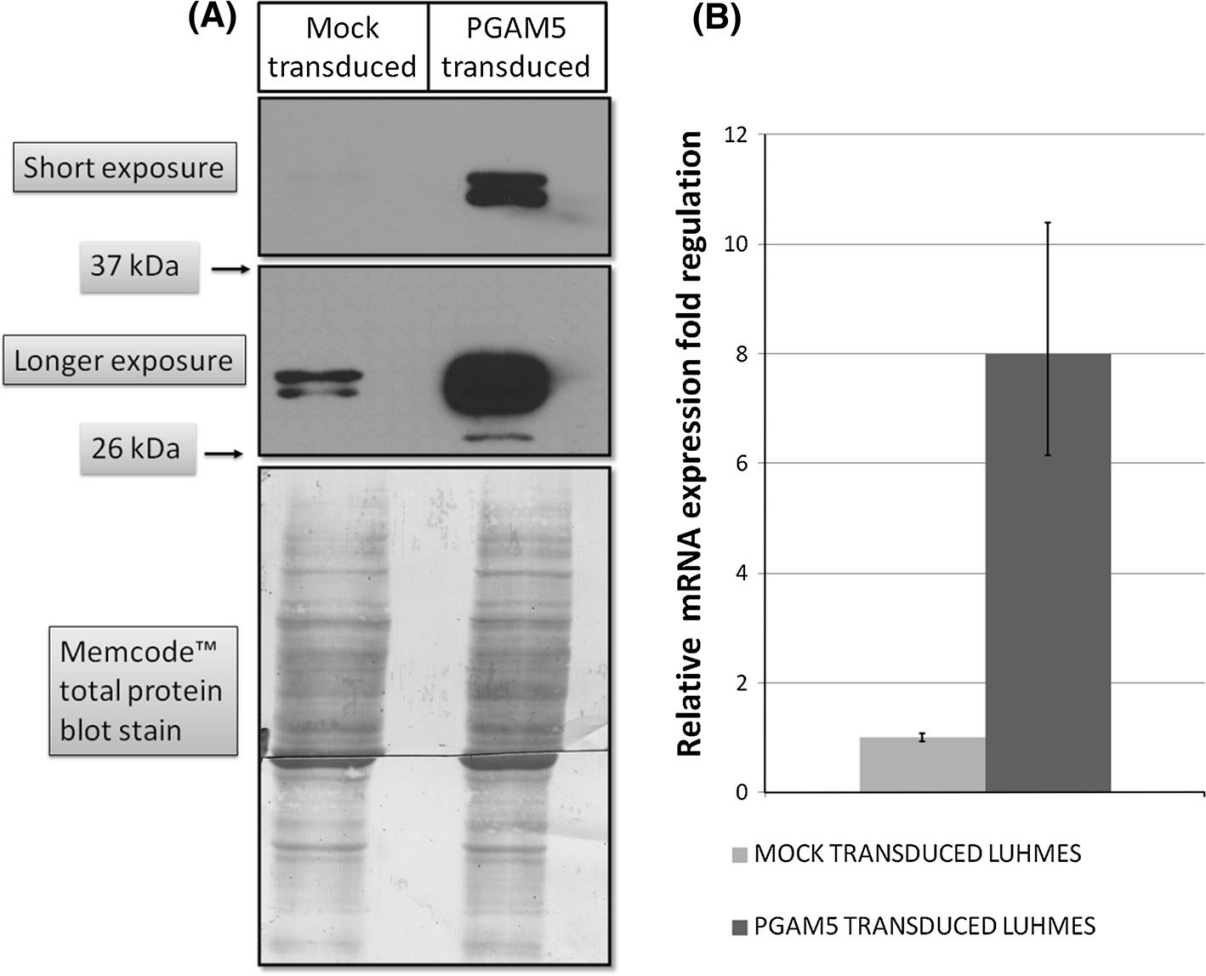
PGAM5 expression in lentivirally transduced LUHMES cells. **a** The abundance of PGAM5_L protein was analyzed by Western Blot. **b** The overexpression of PGAM5_L mRNA was quantified in Real-Time PCR. Error bars represent SD of fold change calculated from three independent experiments

LUHMES cells were transduced at the first day of differentiation with efficiency around 90 %, as estimated by DsRed2 fluorescence. Overexpression of PGAM5 long isoform was confirmed by Real-Time PCR and Western Blot. The *PGAM5_L* mRNA was 8 times more abundant in transduced LUHMES cells (Fig. 2b). Antibody against PGAM5_L protein detected two bands around 32 kDa, which were identified as PGAM5 long isoform and the product of its cleavage by PARL protease (Sekine et al. 2012; Wang et al. 2012). In PGAM5_L overexpressing cells the PARL-proteolytic form was more abundant than intact protein. Conversely, in mock-transduced cells the protein band attributed to intact protein was more intense than the band characteristic for proteolytic PGAM5 form. Furthermore, an additional band around 28–30 kDa was detected in lysates of PGAM5_L overexpressing cells suggesting the existence of an unknown smaller proteolytic form (Fig. 2a).

**Fig. 3:**
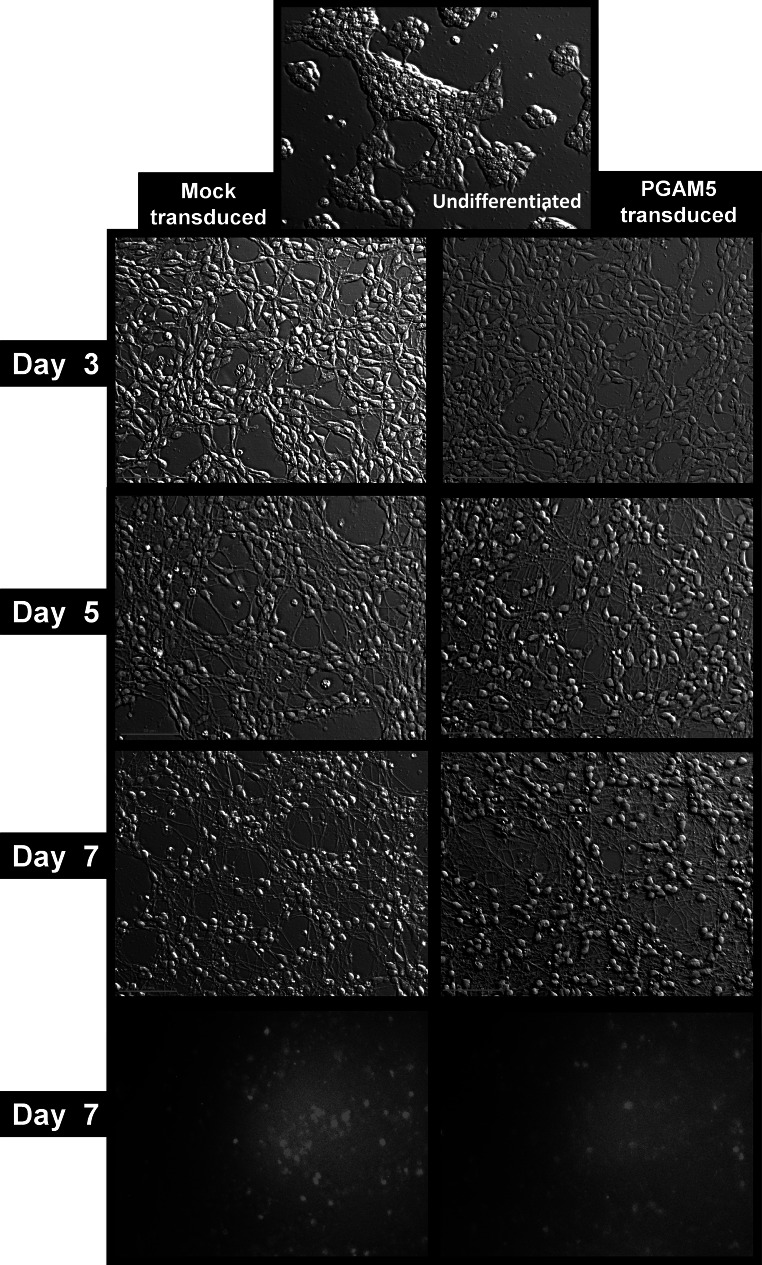
LUHMES cells morphology during differentiation is not influenced by PGAM5 transduction. Pictures of LUHMES cells, mock or PGAM5_L transduced, were taken in ×20 magnitude in Hoffman modulation contrast at third, fifth and seventh day of differentiation. Same cells were analyzed for red fluorescence of DsRed2 (the marker of transduction)

Daily live cell imaging revealed that neither PGAM5_L overexpression nor introduction of mtDsRed2 had any effect on LUHMES cells differentiation or cell morphology. Both, mtDsRed2 transduced (mock transduction) and PGAM5_L overexpressing cells (mtDsRed2 + PGAM5_L) uniformly changed their phenotype to typical neuronal shape (Figs. 1, 3). We also observed no differences when we compared the pattern of wild type cells differentiation with transductants differentiation (data not shown). Neurite outgrowth started to be visible at the third day of differentiation and after 5 day cells created a highly interconnected and multi-branched “neuronal net” as described by a previously established protocol (Scholz et al. 2011).

### 6-OHDA Treatment, But not the PGAM5_L Overexpression, cause Profound Changes in the Expression of PD-Related Genes

We assayed the expression of 84 genes directly or potentially involved in PD etiology. All the genes included in the analysis are connected with PD development or their expression changed in various animal models of PD.

The analysis revealed no statistically significant differences in PD-related gene expression between the mock transduced and PGAM5_L overexpressing cells. Consequently, only small, statistically not significant changes in gene expression were observed between mock transduced and PGAM5_L overexpressing cells treated with 6-OHDA. Nevertheless, the treatment of LUHMES cells with 6-OHDA significantly affected PD-related genes expression. As PGAM5 overexpression did not affect 6-OHDA-induced gene expression, to calculate 6-OHDA-induced gene expression changes the results of both groups (PGAM5 and mock transduced) were pooled to further strengthen the statistical significance of fold regulation levels.

Among 84 assayed genes, the expression of 44 changed more than 20 % (*p* ≤ 0.02), 11 were up-regulated and 33 were down-regulated. Among those, the expression of 20 genes changed more than 200 % (*p* ≤ 0.020) (Table 1). Considering functional gene groups assayed, we did not observe the gene expression changes in genes related to inflammation. The most abundant gene expression changes were observed for genes related to: mitochondria, ion transport, cell adhesion, and interconnection/vesicle signaling (Table 2).

**Table 1:** PD-related genes expression fold change in the LUHMES cells treated with 100 μM 6-OHDA

Gene name	Gene symbol	Aliases	Student`s *t* test *p* value	Fold regulation
*Serine/arginine-rich splicing factor 7*	*SRSF7*	*9G8, AAG3, SFRS7*	*p* < 10^−9^	**2,272407525**
Adenomatous polyposis coli	*APC*	*BTPS2, DP2, DP2.5, DP3, GS, PPP1R46*	*p* < 10^−8^	**-3,515342822**
Phosphatase and tensin homolog	*PTEN*	*10q23del, BZS, CWS1, DEC, GLM2, MHAM, MMAC1, PTEN1, TEP1*	*p* < 10^−8^	**-2,324764994**
N-ethylmaleimide-sensitive factor	*NSF*	*SKD2*	*p* < 10^−7^	**-2,453703041**
Cell division cycle 27 homolog (S. cerevisiae)	*CDC27*	*ANAPC3, APC3, CDC27Hs, D0S1430E, D17S978E, HNUC, NUC2*	*p* < 10^−7^	**-1,992708428**
Cell division cycle 42 (GTP binding protein, 25 kDa)	*CDC42*	*CDC42Hs, G25 K*	*p* < 10^−7^	**-1,534069252**
Ubiquitin-like modifier activating enzyme 1	*UBA1*	*A1S9, A1S9T, A1ST, AMCX1, GXP1, POC20, SMAX2, UBA1A, UBE1, UBE1X*	*p* < 10^−7^	**-1,595294085**
Cullin 2	*CUL2*	–	*p* < 10^−6^	**-2,3082953**
Optic atrophy 1 (autosomal dominant)	*OPA1*	*MGM1, NPG, NTG, largeG*	*p* < 10^−6^	**-1,780188065**
Actin, beta	*ACTB*	*BRWS1, PS1TP5BP1*	*p* < 10^−6^	**-1,562033576**
*Vesicle-associated membrane protein 1 (synaptobrevin 1)*	*VAMP1*	*SYB1, VAMP-1*	*p* < 10^−6^	**5,010440698**
PTEN-induced putative kinase 1	*PINK1*	*BRPK, PARK6*	*p* < 10^−6^	**-1,507431572**
Amyloid beta (A4) precursor protein	*APP*	*AAA, ABETA, ABPP, AD1, APPI, CTFgamma, CVAP, PN-II, PN2*	*p* < 10^−6^	**-1,745533857**
Reticulon 1	*RTN1*	*NSP*	*p* < 10^−6^	**-1,502605119**
*Egl nine homolog 1 (C. elegans)*	*EGLN1*	*C1orf12, ECYT3, HIF-PH2, HIFPH2, HPH-2, HPH2, PHD2, SM20, ZMYND6*	*p* < 10^−6^	**1,525649248**
Heat shock 70 kDa protein 4	*HSPA4*	*APG-2, HS24, P52, HSPH2, RY, hsp70, hsp70RY*	*p* < 10^−5^	**-2,832602738**
G protein-coupled receptor 37 (endothelin receptor type B-like)	*GPR37*	*EDNRBL, PAELR, hET(B)R-LP*	*p* < 10^−5^	**-1,986643383**
Ca ++-dependent secretion activator	*CADPS*	*CADPS1, CAPS, CAPS1*	*p* < 10^−5^	**-2,981895566**
Caspase 3, apoptosis-related cysteine peptidase	*CASP3*	*CPP32, CPP32B, SCA-1*	*p* < 10^−5^	**-2,03831021**
Voltage-dependent anion channel 3	*VDAC3*	*HD-VDAC3, VDAC-3*	*p* < 10^−5^	**-1,494780659**
Parkinson protein 2, E3 ubiquitin protein ligase (parkin)	*PARK2*	*AR-JP, LPRS2, PDJ, PRKN*	*p* < 10^−4^	**-2,369409925**
S-phase kinase-associated protein 1	*SKP1*	*EMC19, OCP-II, OCP2, SKP1A, TCEB1L, p19A*	*p* < 10^−4^	**-1,4349779**
Synaptotagmin I	*SYT1*	*P65, SVP65, SYT*	*p* < 10^−4^	**-2,755530058**
Parkinson protein 7	*PARK7*	*DJ-1, DJ1, HEL-S-67p*	*p* < 10^−4^	**-1,575583282**
Ataxin 3	*ATXN3*	*AT3, ATX3, JOS, MJD, MJD1, RP11-529H20.5, SCA3*	*p* < 10^−4^	**-1,782955859**
Glucan (1,4-alpha-), branching enzyme 1	*GBE1*	*APBD, GBE, GSD4*	*p* < 10^−3^	**-1,685484474**
F-box protein 9	*FBXO9*	*FBX9, NY-REN-57, VCIA1, dJ341E18.2*	*p* < 10^−3^	**-1,530293431**
*Cadherin 8, type 2*	*CDH8*	*Nbla04261*	*p* < 10^−3^	**2,737917368**
Synuclein, alpha (non A4 component of amyloid precursor)	*SNCA*	*NACP, PARK1, PARK4, PD1*	*p* < 10^−3^	**-2,93448938**
Synaptic vesicle glycoprotein 2B	*SV2B*	*HsT19680*	*p* < 10^−3^	**-2,272650385**
*Dopamine receptor D2*	*DRD2*	*D2DR, D2R*	*p* < 10^−3^	**1,520682705**
Delta-like 1 homolog (Drosophila)	*DLK1*	*DLK, DLK-1, Delta1, FA1, PREF1, Pref-1, ZOG, pG2*	*p* < 10^−3^	**-2,493362532**
Regulator of G-protein signaling 4	*RGS4*	*RGP4, SCZD9*	*p* < 10^−3^	**-1,823980398**
*S100 calcium binding protein B*	*S100B*	*NEF, S100, S100-B, S100beta*	*p* < 10^−3^	**5,504391369**
*CXXC finger protein 1*	*CXXC1*	*2410002I16Rik, 5830420C16Rik, CFP1, CGBP, HsT2645, PCCX1, PHF18, SPP1, ZCGPC1, hCGBP*	*p* < 10^−3^	**1,54484259**
*Caspase 8, apoptosis-related cysteine peptidase*	*CASP8*	*ALPS2B, CAP4, Casp-8, FLICE, MACH, MCH5*	*p* < 10^−2^	**3,087237999**
*Aldehyde dehydrogenase 1 family, member A1*	*ALDH1A1*	*ALDC, ALDH-E1, ALDH1, ALDH11, HEL-S-53e, HEL12, PUMB1, RALDH1*	*p* < 10^−2^	**2,151296669**
Potassium inwardly-rectifying channel, subfamily J, member 6	*KCNJ6*	*BIR1, GIRK-2, GIRK2, KATP-2, KATP2, KCNJ7, KIR3.2, hiGIRK2*	*p* < 10^−2^	**-1,526711915**
*Trophoblast glycoprotein*	*TPBG*	*5T4, 5T4AG, M6P1*	*p* < 10^−2^	**2,045570763**
Dopa decarboxylase (aromatic L-amino acid decarboxylase)	*DDC*	*AADC*	*p* < 10^−2^	**-2,037636174**
*Caspase 1, apoptosis-related cysteine peptidase (interleukin 1, beta, convertase)*	*CASP1*	*ICE, IL1BC, P45*	*p* < 0,02	**1,993516068**
Neurofilament, light polypeptide	*NEFL*	*CMT1F, CMT2E, NF-L, NF68, NFL*	*p* < 0,02	**-1,225922474**
Transcription factor 7-like 2 (T cell specific, HMG-box)	*TCF7L2*	*TCF-4, TCF4*	*p* < 0,02	**-1,462013976**
Solute carrier family 18 (vesicular monoamine), member 2	*SLC18A2*	*SVAT, SVMT, VAT2, VMAT2*	*p* < 0,02	**-1,390565564**

Over expressed gene names are highlighted in italic font. Statistical differences were examined by Student’s t test with *p* < 0.02 considered to be statistically significant. Only statistically significant gene expression changes higher than 20 % are shown. The *p* values and fold regulation levels were calculated from six experiments

**Table 2:** The functional groups of PD-related genes and their expression regulation in response to 6-OHDA

Functional gene group	Genes belonging to the group and their expression regulation in response to 6-OHDA
Parkin complex	HSPA4 (HSP70), PARK7, *STUB1*
Parkin Substrate	*ATXN2*, ATXN3, GPR37, *SYT11*
Cell adhesion	APC, APP, **CDH8**, *FN1, NFASC, NRXN3*, PTEN, **TPBG**.
Ubiquitination	CDC27, CUL2, FBXO9, *LRRK2, PAN2, PARK2*, PINK1, SKP1, *STUB1, UBB*, UBA1, *UBE2I, UBE2 K, UBE2L3, UCHL1, USP34*
Inflammation	*FN1, PRDX2, YWHAZ*
Apoptosis	APC, APP, **CASP1 (ICE)**, CASP3, **CASP8 (FLICE)**, *CASP9, CUL2, MAPK9 (JNK2), PSEN2*, PTEN, *BDNF, CASP3*, NEFL, *NR4A2 (NURR1)*, OPA1, *PPID, PRDX2, PSEN2, SLC25A4*, SNCA, TCF7L2, *UBB, YWHAZ*
Mitochondria	CASP3, *CASP7*, **CASP8 (FLICE)**, *HSPA4 (HSP70), LRRK2*, NEFL, OPA1, PARK7, PINK1, PTEN, *SLC25A4*, SNCA, *TH, UCHL1*, **VAMP1**, VDAC3, *YWHAZ*
Synaptic Vesicles	*LRRK2, SEPT5*, SV2B, *SYNGR3*, SYT1, *SYT11, TH*
Signal Transduction	Dopaminergic: *NSG1*, DDC, **DRD2**, *HTR2A, NR4A2 (NURR1), PARK2*, PARK7, PINK1, *SEPT5, SLC6A3*, SNCA, *TH*
	GABAergic: **DRD2**, *GABBR2* MAP Kinase: APC, *FGF13, MAPK9 (JNK2), PRDX2*, RGS4
	Notch: APP, *PSEN2, SPEN*
Cytoskeletal organization	APC, CDC42, *MAPT*, NEFL, *PARK2*.
Ion transport	*ATP2B2*, CADPS, **CXXC1, DRD2, EGLN1**, *GBE1, GRIA3, HTR2A*, KCNJ6, NSF, *PSEN2*, **S100B, SRSF7**, *SLIT1*, SNCA, VDAC3
Transporters	*ATP2B2, GRIA3*, SLC18A2, *SLC6A3, SLC25A4*, SV2B, SYT1, *SYT11*, VDAC3
Others	**ALDH1A1**, *BASP1, CHGB*, DLK1, *NCOA1, NTRK2, RTN1*

Up-regulated genes shown in bold. Down-regulated genes shown in undeline. Tested genes whose expression was not changed are highlighted in italic

## Discussion

It was previously shown that PGAM5 regulates mitophagy in response to mitochondria depolarizing agent CCCP or hypoxia, by two distinct mechanisms (Sekine et al. 2012; Wu et al. 2014). Very recently, *Pgam5* knockout mice were analyzed and PGAM5 role in mitophagy induction was established in an animal model. Mice lacking *Pgam5* gene were characterized by a PD like movement disorder, which is thought to be mediated by a dopaminergic neurons degeneration due to an inefficient mitophagy (Lu et al. 2014). Thus, it was of interest to elucidate whether PGAM5 overexpression would affect the action of the model PD-inducing toxin, 6-OHDA.

While the Pgam5 deficiency resulted in the induction of PD resembling disorder in knock out mice, over expression of the protein in differentiated neurons (LUHMES cells) did not change the gene expression of any of the tested 84 PD-related genes, neither in vehicle-treated control nor in 100 μM 6-OHDA-treated cells. It is plausible that PGAM5 post translation modifications are more important for mediating its biological role than its abundance or up-regulation does not have as pronounced biological effects as its knockdown.

mtDsRed2 was introduced into lentiviral plasmids to act as a transduction control and to allow visualization of mitochondria dynamics. Judging from the mtDsRed2 fluorescence, its expression was relatively low, but high enough to act as a transduction efficiency control. We hypothesize that this could have been caused by P1 promoter silencing and/or promoter or protein coding sequence mutation. Nevertheless, low mtDsRed2 expression was not consistent with change in the expression of PGAM5, which was driven by the different promoter. Due to limited resources and fact that weak mtDsRed2 expression is even beneficial because that strong mtDsRed2 expression might have interfered with biological effects of PGAM5 overexpression, we have not further studied this problem and used mtDsRed2 only as a transduction efficiency control.

*PINK1* and *PARK2* (coding PARKIN E3 ubiquitin ligase) are one of the currently known 12 genes, whose monogenic mutations are found in genomes of patients suffering from early or juvenile onset PD (Bonifati 2014). Those two genes work in a common signaling pathway responsible for triggering selective autophagy of mitochondria and regulating mitochondria transport (Pickrell and Youle 2015; Scarffe et al. 2014). The treatment with 100 μM 6-OHDA caused down regulation of two cytoprotective genes and members of the Parkin complex: *HPS70* and *PARK7* (coding DJ-1 protein). The HPS70 and DJ-1 proteins bind each other to regulate the cellular defense mechanism linked to oxidative insults (Moore et al. 2005). *PARK7* is one of the five known genes whose mutations have been linked to familial forms of PD. DJ-1 is generally considered being cytoprotective, as it is up-regulated during oxidative stress and it is able to rescue cells from apoptosis in response to parkinsonian toxins (Bonifati et al. 2003; Inden et al. 2006; Yokota et al. 2003). In SH-SY5Y neuroblastoma cells DJ-1 is up-regulated in response to 6-OHDA (Lev et al. 2008). Interestingly, in LUHMES cells, we have observed a half-fold decrease in DJ-1 expression (*p* < 10^−4^) and almost 3-fold decrease in HSPA4 (*p* < 10^−5^). This may indicate that LUHMES cells are more sensitive to oxidative insults. Moreover, PINK1 and alpha synuclein, other genes mutated in mendelian forms of PD, were also down-regulated in LUHMES cells in response to 6-OHDA. It seems that the observed down-regulation of cytoprotective genes related to parkin complex is accompanied by the pro-apoptic rather than pro-survival effect. Indeed, we also observed the three-fold up-regulation of the initiator caspase 8.

The most profound changes in gene expression were observed for synaptobrevin 1 coded by *VAMP1* gene (5-fold up-regulation, *p* < 10^−6^) and adenomatous polyposis coli gene (3.5-fold down-regulation *p* < 10^−8^).

The synaptobrevin 1 is a member of the family of vesicle-associated membrane proteins (VAMPs) that are a part of the SNARE complex involved in vesicle fusion during synaptic signaling and autophagy (Shih et al. 2005). Apart from *VAMP1* up-regulation, we have observed the 6-OHDA-induced decrease in expression of three other genes, whose protein products are directly related to synaptic vesicle signaling: synaptotagmin (*SYT1*, −2.7-fold), synaptic vesicle glycoprotein (*SV2B*,−2.2-fold) and calcium-dependent activator protein for secretion (*CADPS*, −2.9-fold). All of those proteins are implicated in the Ca^2+^-dependent exocytosis. Synaptobrevins are known to be targeted and degraded by tetanospasmin and botulinum—neurotoxins produced by anaerobic pathogens from Clostridium sp. (Binz et al. 2010). The serotypes of those neurotoxins are highly specific toward particular orthologues of VAMP proteins present in different regions of the organism (Yamamoto et al. 2012). Brain orthologues VAMP1 and VAMP2 are present in different regions of the rat brain (Ferecskó et al. 2015; Raptis et al. 2005). Mendieta et al. (2012) shown that C- terminal part of tetanus toxin, when administered by muscle injection protected rats from motor symptoms of Parkinson’s disease induced by 6-OHDA. On the other hand, a case study was published describing severe parkinsonian symptoms observed after tetanus vaccination (Reijneveld et al. 1997). In view of our results, it is plausible that the neuroprotective effects of tetanospasmin C-terminal fragment could be at least partially mediated by its hydrolyzing activity against VAMPs, as we found *VAMP1* to be highly up-regulated in response to 6-OHDA in LUHMES cells. Fine tuning of VAMPs in different brain regions by the use of clostridium toxins or their derivates may be in future a promising therapeutical mechanism to treat neurological disorders.

Adenomatous polyposis coli gene (*APC*) is the second gene, which expression profoundly changed in 6-OHDA-treated LUHMES cells (3.5-fold down-regulation *p* < *10*^−*8*^). This gene is mostly known for its tumor suppressing activities, and its mutation is associated with severe pre-malignant syndrome (Groden et al. 1991; Zhang et al. 2010). There is scare evidence of APC implications in Parkinson’s disease, but it was found to be indispensible for the nicotine acetylcholine synapse assembly (Temburni et al. 2004). Furthermore, nicotine is believed to have neuroprotective role against PD, as various epidemiologic studies showed that smokers less frequently developed PD (Hernan et al. 2002; Tanaka et al. 2010).

The decrease in dopamine content in the central nervous system is the main cause of motor symptoms of PD. Our gene expression analysis was also focused on genes related to dopaminergic signaling. We found that 6-OHDA caused a two-fold decrease in the expression of DOPA decarboxylase, an enzyme which catalyzes a rate limiting step of dopamine synthesis. This decrease was accompanied by the 50 % increase in the expression of dopamine receptor 2. The increase in the expression of dopamine receptor 2 is probably a part of the compensation mechanism of dopaminergic neurons, as it has been shown that degeneration of striatal dopaminergic neurons is accompanied by the compensation mechanisms that allow maintaining dopamine concentrations at the unchanged levels (Golden et al. 2013; Zigmond et al. 1990).

The treatment with 100 μM 6-OHDA led also to the 5.5-fold increase in the expression of *S100B* gene. S100B is a calcium binding peptide with plenty of known biological implications. S100 peptide is implicated in regulation of proliferation, differentiation, apoptosis, Ca^2+^ homeostasis, energy metabolism, inflammation, and migration/invasion through interactions with a variety of target proteins including enzymes, cytoskeletal subunits, receptors, transcription factors, and nucleic acids (Donato et al. 2013). S100B is a prototype biomarker for brain injuries including trauma and stroke. We found that in control cells the expression of S100B was very weak, while in 6-OHDA-treated cells it increased to the levels that could be reliably quantified by RT-PCR. S100B is expressed and released by astro and oligodendrocytes in response to glial activation or injury and was recently studied as a potential serum biomarker for traumatic brain injury, stroke, depression, and blood brain barrier injury (Blyth et al. 2009; Schroeter et al. 2013; Vos et al. 2004). S100B is also produced by glia cells in PD associated neuroinflammation (Niranjan 2014). The role of S100B up-regulation in 6-OHDA-treated LUHMES cells remains elusive, but may be involved in the processes of neurite outgrowth and/or cytoprotection as a part of the response to insults causing neurite degeneration, as S100B was attributed as a neurotropic factor (McAdory et al. 1998).

In conclusion for the first time, we presented a detailed analysis of PD-related gene expression in LUHMES cells treated with 6-OHDA and found out that the observed profile of expression was not influenced by the lentiviral overexpression of PGAM5 a protein recently implicated in various processes related to the development of PD. We believe that our results will be of help for researchers working on PGAM5 and Parkinson’s Disease and that they will encourage them to use LUHMES cells that are currently emerging as a modern model for neurological disorders.

## Electronic supplementary material

The map of the plasmid elements of pLV.Des2d.P_mito-DsRed2_PGAM5_L vector (TIFF 26187 kb)

The map of the plasmid elements of pLV.Des2d.P_mito-DsRed2_3xFLAG_MCS vector (TIFF 31061 kb)
