# Supplementary figures and images for: 6-OHDA-Induced Changes in Parkinson`s Disease-Related Gene Expression are not Affected by the Overexpression of PGAM5 in In Vitro Differentiated Embryonic Mesencephalic Cells
Source: Cell Mol Neurobiol. 2015 May 19;35(8):1137–47. doi: 10.1007/s10571-015-0207-5 (PMC4602069; doi:10.1007/s10571-015-0207-5)

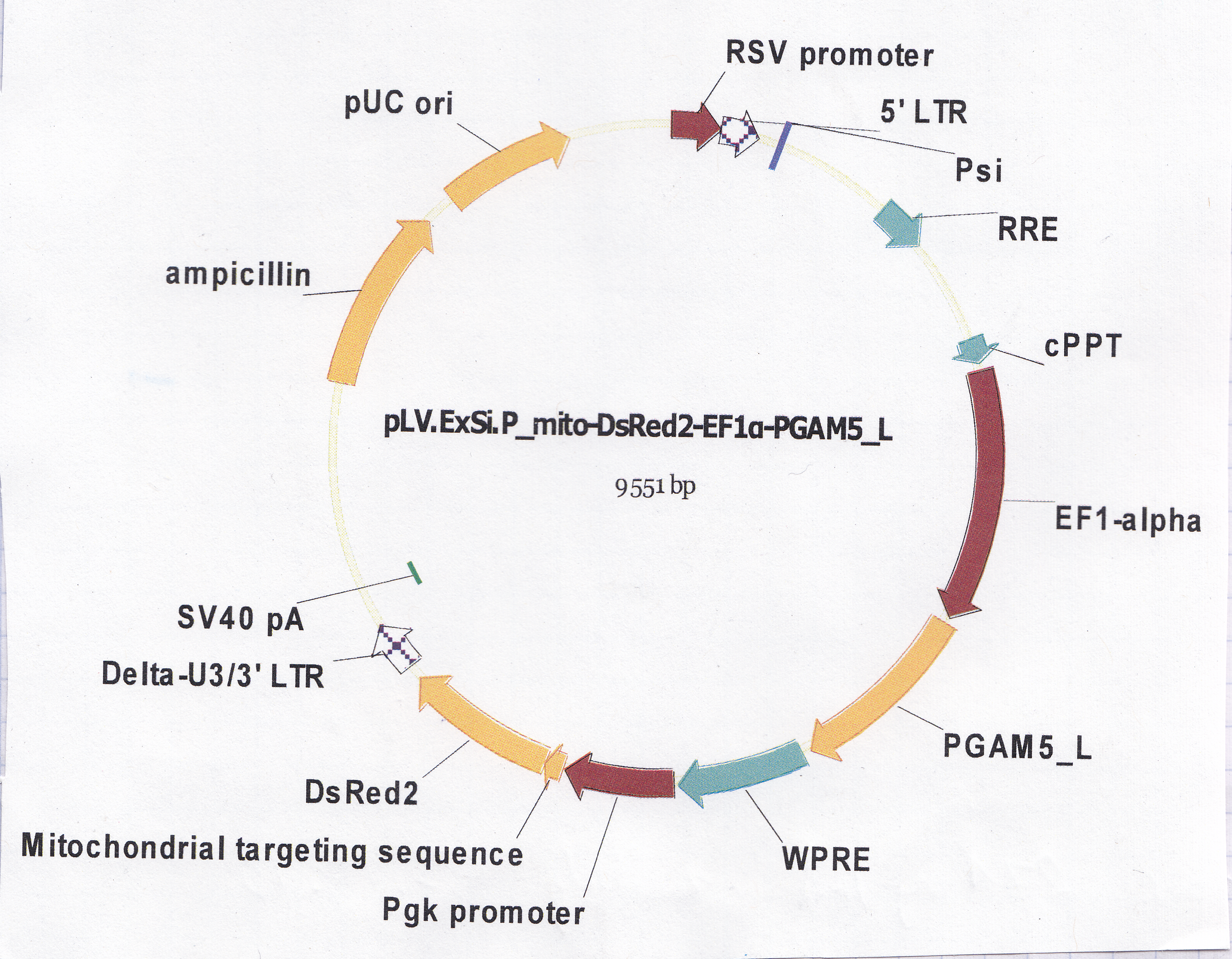

Supplement: Supplementary file 1 — The map of the plasmid elements of pLV.Des2d.P_mito-DsRed2_PGAM5_L vector (TIFF 26187 kb) [file 10571_2015_207_MOESM1_ESM.tif]

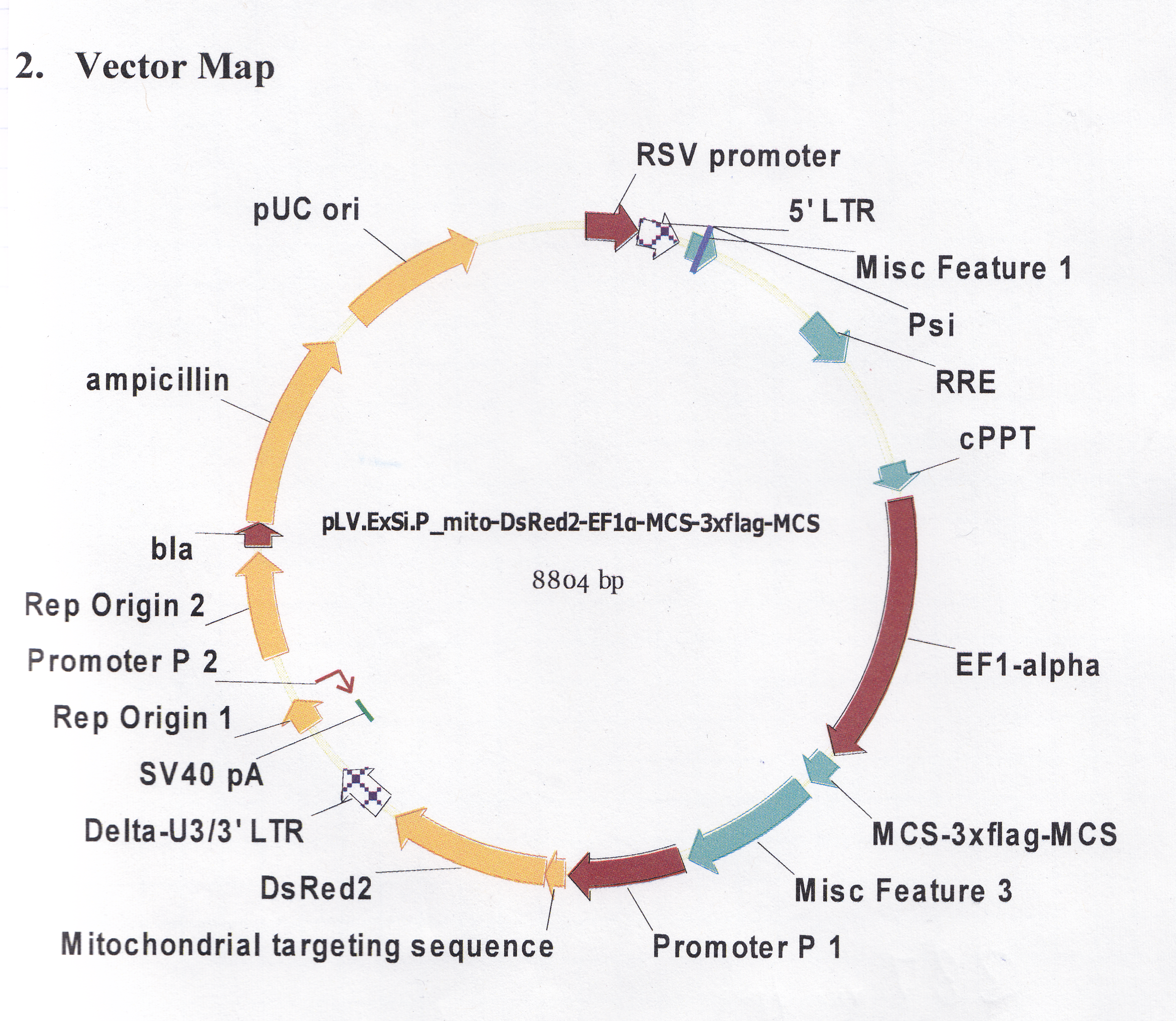

Supplement: Supplementary file 2 — The map of the plasmid elements of pLV.Des2d.P_mito-DsRed2_3xFLAG_MCS vector (TIFF 31061 kb) [file 10571_2015_207_MOESM2_ESM.tif]
